# Carbon monoxide prevents hepatic mitochondrial membrane permeabilization

**DOI:** 10.1186/1471-2121-12-10

**Published:** 2011-03-09

**Authors:** Cláudia SF Queiroga, Ana S Almeida, Paula M Alves, Catherine Brenner, Helena LA Vieira

**Affiliations:** 1 Instituto de Biologia Experimental e Tecnológica (IBET), Apartado 12, 2781-901 Oeiras, Portugal; 2 Instituto de Tecnologia Química e Biológica (ITQB), Universidade Nova de Lisboa, Apt 127, 2781-901, Oeiras, Portugal; 3 INSERM U769 Université Paris-Sud, Faculté de Pharmacie, Châtenay Malabry, France

## Abstract

**Background:**

Low concentrations of carbon monoxide (CO) protect hepatocytes against apoptosis and confers cytoprotection in several models of liver. Mitochondria are key organelles in cell death control *via *their membrane permeabilization and the release of pro-apoptotic factors.

**Results:**

Herein, we show that CO prevents mitochondrial membrane permeabilization (MMP) in liver isolated mitochondria. Direct and indirect approaches were used to evaluate MMP inhibition by CO: mitochondrial swelling, mitochondrial depolarization and inner membrane permeabilization. Additionally, CO increases mitochondrial reactive oxygen species (ROS) generation, and their scavenging, by ß-carotene addition, decreases CO protection, which reveals the key role of ROS. Interestingly, cytochrome c oxidase transiently responds to low concentrations of CO by decreasing its activity in the first 5 min, later on there is an increase of cytochrome c oxidase activity, which were detected up to 30 min.

**Conclusion:**

CO directly prevents mitochondrial membrane permeabilization, which might be implicated in the hepatic apoptosis inhibition by this gaseoustransmitter.

## Background

Carbon monoxide (CO) is usually considered a harmful and toxic molecule due to its high affinity to heme proteins. However, recent evidences show that low doses of CO can be cytoprotective, presenting several biological properties, namely, anti-apoptosis, anti-proliferation, anti-inflammation and vasodilatation [1]. Furthermore, CO is an endogenous product of heme degradation by heme-oxygenase (HO), generating free iron and biliverdin as by-products. In fact, HO system is essential for tissue response to diverse pathological contexts, aiming at restoring and/or maintaining cellular homeostasis [2].

In hepatocytes and/or liver models, CO appears to act as an anti-apoptotic molecule. By stimulating ATP production, CO activates p38 MAPK signalling, preventing apoptosis in human hepatocytes [3]. CO rescues mice from fulminant hepatitis, presenting a marked reduction of TNF-alpha-induced apoptosis [4] or *via *NO generation [5]. In primary cultures of rat hepatocytes, CO limits cytotoxicity induced by glucose deprivation through suppression of ERK MAPK activation [6]. In an endotoxic shock model, CO protects hepatocytes from apoptosis by augmenting iNOS expression [7]. It is also described that superoxide anion-induced apoptosis is inhibited by CO *via *limiting JNK activity [8]. CO treatment protects hepatocytes from cell death by inducing NF-kB activation, which is dependent on ROS generation, since inhibition of ROS generation (via anti-oxidant addition or by using respiratory deficient cells) reverses CO-induced cytoprotection [9]. Among all publications showing CO as anti-apoptotic molecule in hepatic model, only Kim and colleagues [10] have mentioned the involvement of mitochondria. CO protects hepatocytes from TNF-alpha/Actinomycin D-induced apoptosis by activating NF-kB, which is associated with a reduction in cytochrome c release from mitochondria [10]. However, no data demonstrate the direct role of CO into isolated liver mitochondria.

Mitochondria play a key role in the intrinsic pathways of apoptosis. Many pro-apoptotic factors are confined in the inter-membrane space, and upon mitochondrial membrane permeabilization (MMP) these factors are released into the cytosol and cell death becomes an irreversible process [11]. MMP marks a point of no return in the apoptotic intrinsic pathways by activating both caspase-dependent and caspase-independent mechanisms. The rupture of mitochondrial membrane also leads to the functional impairment of mitochondria, bioenergetic and redox crisis with ATP depletion and strong oxidative stress [12]. Therefore, mitochondria become a crucial target to modulate cell death in several models.

Based on the following facts: (i) CO is an anti-apoptotic molecule in several hepatic models, hepatocytes and/or liver and (ii) mitochondria are central executers of cell death process, *via *the mitochondrial membrane permeabilization (MMP); we explored the direct effect of CO into isolated liver mitochondria (MMP modulation) and the involvement of ROS in this process. MMP was assessed by mitochondrial depolarization, inner membrane permeabilization and mitochondrial swelling.

## Results

### Assessment of CO toxicity and establishment of optimal CO concentration in isolated liver mitochondria

**Figure 1:**
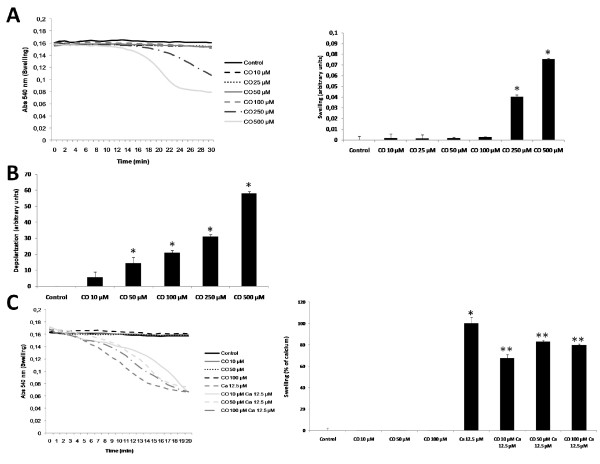
**Effect of carbon monoxide concentration in mitochondrial swelling**. Mitochondrial swelling was followed by the decrease in absorbance at 540 nm at 37°C for 30 minutes. **A**. Mitochondrial swelling was assessed in the presence of CO at 0, 10, 25, 50, 100, 250 and 500 μM. *Left panel*, representative graphics of experiments, performed in triplicates and repeated, at least, three times. *Right panel*, quantitative expression of mitochondrial swelling measured at 28 minutes of incubation. All values are mean ± SD (error bars), n = 3; *p < 0.05, compared to control mitochondria. **B**. Rhodamine 123 fluorescence change (λ_exc_: 485 nm, λ_em_: 535 nm) was used to measure mitochondrial depolarization and to test CO toxicity in isolated liver mitochondria. Quantitative expression of mitochondrial depolarization measured at 15 minutes after CO treatment, all values are mean ± SD (error bars), n = 3; *p < 0.05, compared to non-treated mitochondria. **C**. Mitochondria were pre-treated with CO at 0, 10, 50 or 100 μM for 15 minutes at RT, and measurements were acquired at 37°C after Ca^2+ ^at 12.5 μM addition to trigger swelling. *Left panel, r*epresentative graphics of experiments, which were done in triplicates and repeated, at least, three times. *Right panel*, quantitative expression of mitochondrial swelling measured at 12 minutes of incubation. The effect of 12.5 μM of Ca^2+ ^was normalized as 100% of swelling. All values are mean ± SD (error bars), n = 3; *p < 0.05, compared to control mitochondria; **p < 0.05, compared to Ca^2+ ^12.5 μM treated mitochondria.

In order to evaluate the toxicity of carbon monoxide (CO) on isolated liver mitochondria, swelling and depolarization assays were performed with different doses of CO (10 to 500 μM). Up to 100 μM and for 30 minutes, CO is not able to trigger mitochondrial swelling. However CO triggers swelling at concentrations of 250 and 500 μM (Figure 1A), indicating that at higher concentrations this gas induces mitochondrial damage. Because mitochondrial depolarization is an earlier event compared to swelling, lower concentrations of CO have induced lost of ΔΨm, only at 10 μM there is no depolarization induction (Figure 1B). Pre-treatment with 10, 50 or 100 μM of CO for 15 minutes partially prevents mitochondrial swelling induced by calcium (Figure 1C). Since 10 μM presents the highest protection and does not induce mitochondrial depolarization, this concentration is the optimal one to be used in all other assays.

### CO inhibits mitochondrial membrane permeabilization (MMP) in isolated liver mitochondria

**Figure 2:**
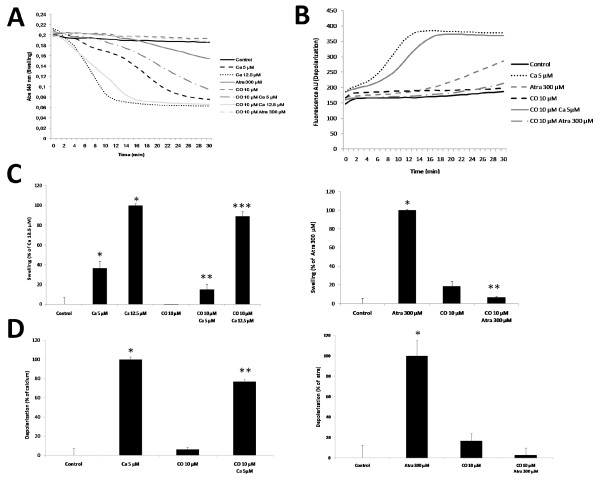
**Carbon monoxide prevents mitochondrial swelling and depolarization**. **A**. Mitochondria were pre-treated with 10 μM of CO for 15 minutes at RT, swelling measurements were performed after 6.25 μM and 12.5 μM of Ca^2+ ^or 300 μM of atractyloside addition into isolated mitochondria. The decrease on absorbance was assessed at 540 nm, at 37°C for 30 minutes and is presented as representative graphics of experiments, which were done in triplicates and repeated, at least, three times. **B**. Mitochondrial depolarization was assessed by rhodamine 123 fluorescence change (λ_exc_: 485 nm, λ_em_: 535 nm), at 37°C, for 30 min, in the absence or presence of CO at 10 μM and atractyloside at 300 μM or Ca^2+ ^at 6.25 or 12.5 μM. **C**. *left panel *Quantitative expression of mitochondrial swelling measured at 15 minutes of incubation and *right panel*, 28 minutes of incubation. The effect of 12.5 μM of Ca^2+ ^(*left panel*) or Atra 300 μM (*right panel*) was normalized as 100% of swelling. All values are mean ±SD (error bars), n = 3; *p < 0.05, compared to control mitochondria; **p < 0.05, compared to Ca^2+ ^6.25 μM (*left panel*) or Atra 300 μM (*right panel*) treated mitochondria; ***p < 0.05, compared to Ca^2+ ^treated mitochondria. **D**. Quantification of mitochondrial depolarization experiments performed in triplicates and repeated, at least, three times. *left panel*, quantitative expression of mitochondrial depolarization measured at 15 minutes of incubation and *right panel*, 28 minutes of incubation. The effect of 6.25 μM of Ca^2+ ^(*left panel*) or Atra 300 μM (*right panel*) was normalized to 100% of swelling. All values are mean ± SD (error bars), n = 3; *p < 0.05, compared to control mitochondria; **p < 0.05, compared to Ca^2+ ^6.25 μM (*left panel*) or Atra 300 μM (*right panel*) treated mitochondria.

**Figure 3:**
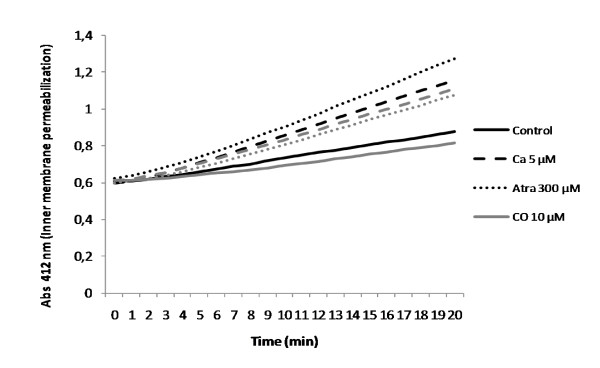
**Carbon monoxide prevents inner membrane permeabilization**. Citrate synthase activity assay was used to follow inner membrane permeabilization. Measurements were performed at 412 nm in the absence or presence of 10 μM of CO and 5 μM of Ca^2+ ^or 300 μM of atractyloside, at 37°C for 20 minutes. Representative graphics of experiments, which were done in triplicates and repeated, at least, three times are shown.

CO partially inhibits mitochondrial swelling (Figure 2A) when liver isolated mitochondria were exposed to 10 μM of CO during 15 minutes at room temperature prior to addition of calcium to induce MMP (Figure 2A and 2C, *left panel*) and of atractyloside, a ligand of ANT that prevents ADP/ATP translocation and induces its pore forming function (Figure 2A and 2C, *right panel*) [13-15]. Swelling quantification analysis was performed at different time points for calcium or atractyloside because of their different mode of action and kinetics (Figure 2C, *left *and *right panel*). Loss of ΔΨm, or mitochondrial depolarization, induced by atractyloside or calcium was also prevented by prior addition of CO at 10 μM (Figure 2B) and quantification analysis was done (Figure 2D) for calcium (*left panel*) and for atractyloside (*right panel*). Changes in the inner membrane permeability (the opening of a large channel for molecules up to ~800 Da) were assessed by an enzymatic assay based on the accessibility of citrate synthase, which is a soluble matrix enzyme [16]. The atractyloside induction of inner membrane permeabilization is partially prevented by CO (Figure 3). Due to the nature of this enzymatic assay, the quantification of the effect has to be done by calculating the slopes of the different curves over time, which were normalised relatively to Ca^2+ ^5 μM (Table 1). CO pre-treatment decreases the slope compared to Ca^2+ ^5 μM (85% *vs *100%) and Atra 300 μM (81% *vs *145%). This reduction demonstrates CO delay in the induction of inner membrane permeabilization. Taken together, CO acts directly on mitochondria in order to limit their membrane permeabilization, shown by its prevention of swelling, depolarization and inner membrane permeabilization.

**Table 1:** Carbon monoxide prevents inner membrane permeabilization

	Control	Ca^2+ ^5 μM	Atra 300 μM	CO 10 μM	CO 10 μM Ca^2+ ^5 μM	CO 10 μM Atra 300 μM
**Slope (Abs/min)**	0,00E + 00	1,57E - 02	2,28E - 02	0,00E + 00	1,34E - 02	1,27E - 02
**% ΔSlope (Abs/min) relative to Ca^2+^**	0,00	100,00 ± 1,02	145,13 ± 1,20	0,00	85,44 ± 0, 53	81,25 ± 0, 46

Inner membrane permeabilization is followed by absorbance at 412 nm. The slope was calculated between 10 to 20 minutes for each different condition in order to assess the effect of CO in the inner membrane permeabilization after treatment with Ca^2+ ^or atractyloside. For slope calculation, absorbance values for each different condition were normalized by the absorbance value corresponding to the control non-treated mitochondria. The difference between control mitochondria and Ca^2+ ^-treated mitochondria slopes correspond to an increase of 100%.

### ROS are important molecules for CO prevention of MMP in liver mitochondria

**Figure 4:**
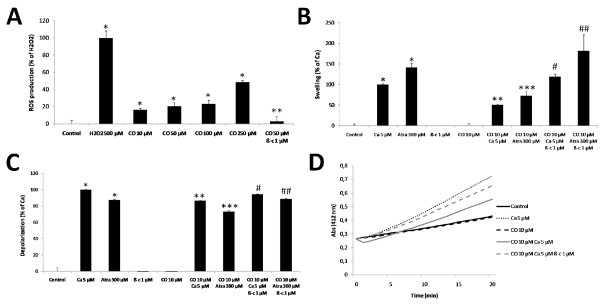
**Influence of ROS on CO effect at mitochondrial level**. **A**. Mitochondria were pre-treated or not with β-carotene, followed by CO at 0, 10, 50, 100 or 250 μM or H_2_O_2 _at 500 μM treatment. ROS quantification using 2',7'-dichlorofluorescein diacetate (H_2_DCFDA, λ_exc_: 485 nm, λ_em_: 530 nm) was done in isolated mitochondria. The values are expressed in relative percentage of 500 μM of H_2_O_2 _(100%) at 28 minutes after treatment. All values are mean ± SD, n = 3; * *p *< 0.05 compared to control mitochondria and ** *p *< 0.05 compared to mitochondria treated with CO at 50 μM. Mitochondria were pre-treated in the presence or absence of 1 μM of β-carotene and/or 10 μM of CO, then atractyloside at 300 μM or calcium at 5 μM was added, followed by swelling measurements (absorbance at 540 nm) for **B **and depolarization measurements (Rhodamine 123, λ_exc_: 485 nm, λ_em_: 535 nm) for **C**. In **B **and **C **the values are expressed in relative percentage of 5 μM of Ca^2+ ^(100%) measured at 15 minutes after treatment. All values are mean ± SD, n = 3; * *p *< 0.05 compared to control mitochondria, ** *p *< 0.05 compared to Ca^2+^-treated mitochondria, *** *p *< 0.05 compared to Atra-treated mitochondria, # *p *< 0.05 compared to β-c and Ca^2+^-treated mitochondria and ## *p *< 0.05 compared to β-c and Atra-treated mitochondria. **D**. Inner membrane permeabilization was assessed according to [16]. Mitochondria were pre-treated 1 μM of β-carotene and 10 μM of CO, followed by addition of calcium at 5 μM. Data was acquired at 412 nm for 20 minutes at 37°C.

Mitochondrial generated reactive oxygen species (ROS) are described as imperative signalling molecules for CO biological functions [17-20]. Thus, the role of ROS in CO-induced protection in isolated liver mitochondria was also evaluated. First, it was verified whether CO increases ROS generation in isolated liver mitochondria in a dose-response manner and this generation is limited by pre-treatment with ß-carotene (Figure 4A). To disclose ROS role in this system, their level augmentation was prevented by treating mitochondria with ß-carotene prior to CO addition and MMP induction. Indeed, CO inhibition of mitochondrial swelling (Figure 4B), depolarization (Figure 4C) and inner membrane permeabilization (Figure 4D and Table 2) were, at least, partially prevented by the anti-oxidant ß-carotene. Thus, at mitochondrial level, ROS appear to be important signalling molecules for CO mode of action during inhibition of mitochondrial membrane permeabilization.

**Table 2:** Role of ROS in CO prevention of inner membrane permeabilization

	Control	Ca^2+ ^5 μM	CO 10 μM	CO 10 μM Ca^2+ ^5 μM	CO 10 μM Ca^2+ ^5 μM ß-c 1 μM
**Slope (Abs/min)**	0,00E + 00	1,78E - 02	00E00	9,60E - 03	1,34E - 02
**% ΔSlope (Abs/min) relative to Ca^2+^**	0,00	100,00 ± 0,68	0,00	53,79 ± 0,56	75,22 ± 0,65

Inner membrane permeabilization is followed by absorbance at 412 nm. The slope was calculated between 10 to 20 minutes for each different condition in order to assess the effect of CO in the inner membrane permeabilization in the presence or absence of ß-carotene and after treatment with Ca^2+^. For slope calculation, absorbance values for each different condition were normalized by the absorbance value corresponding to the control non-treated mitochondria. The difference between control mitochondria and Ca^2+ ^-treated mitochondria slopes correspond to an increase of 100%.

### Low concentrations of CO transiently prevents cytochrome c oxidase (COX) activity

**Figure 5:**
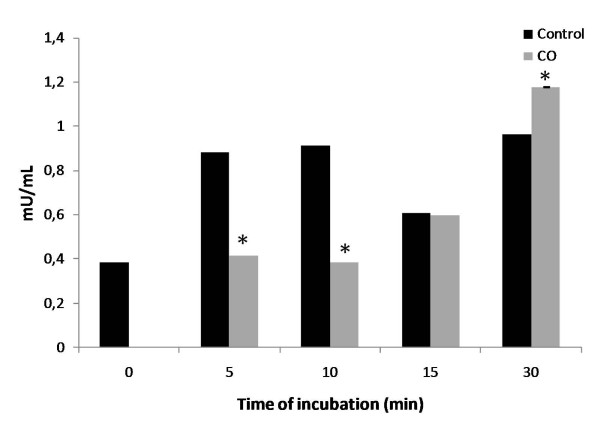
**Carbon monoxide effect on cytochrome c oxidase activity**. COX activity determinations were performed at 550 nm, for 1 minute at 25°C, after a treatment with 10 μM of CO at 37°C for 5, 10, 15 and 30 minutes. For each sample, COX activity was determined and expressed in mU/mL. All values are mean ± SD, n = 3; * *p *< 0.05 compared to control mitochondria.

Major intracellular sources of ROS are the oxidative phosphorilation complexes. Moreover, accordingly to the literature, CO is described to prevent cytochrome c oxidase (COX) activity [17]. Thus, CO direct effect on the COX activity was assessed in isolated liver mitochondria. CO at 10 μM transiently prevents COX activity up to 10 minutes, while 30 minutes later the effect is inverted; CO slightly accelerates COX activity (Figure 5).

## Discussion

Carbon monoxide has been described to be involved in protection of hepatocytes against cell death. Kim and colleagues [10] have demonstrated that CO decreases Bcl-2 family proteins translocation into mitochondria, limiting cytochrome c release into the cytosol [10]. Despite the crucial role of mitochondria in cell death control and the potent anti-apoptotic property of CO in hepatocytes, the direct effect of CO in isolated liver mitochondrial membrane permeabilization has never been reported before. Herein it is shown that low doses of this gaseous molecule prevent mitochondrial membrane permeabilization. Recently, we have shown that CO limits mitochondrial membrane permeabilization in non-synaptic mitochondria isolated from rat brain cortex [21], which was accompanied by inhibition of cytochrome c release from mitochondria and by glutathionylation of adenine nucleotide translocase (ANT). In this cerebral model, CO protects astrocytes against cell death and ROS generation appears to be important for this pathway [21]. Furthermore, in other systems, it is generally recognized that several CO biological functions are dependent on mitochondrial ROS generation and signalling [17-21]. In the present work, ROS also emerge as significant molecules involved in the signal transduction at the mitochondrial sub-cellular level. Inhibition of mitochondrial ROS generation by an anti-oxidant addition (ß-carotene) reverses CO prevention of liver mitochondrial membrane permeabilization (Figure 4), which confirms their key role. Still one might hypothesize that ROS promote post-translational modifications on mitochondrial proteins, as described for ANT glutathionylation in non-synaptic mitochondria [21].

The most accepted hypothesis for CO-induced mitochondrial ROS production is *via *partial inhibition of cytochrome c oxidase, accumulating electrons at complex III level. The generated anion superoxide is rapidly converted into hydrogen peroxide [17]. According to our results, and using low concentrations of CO (10 μM), COX inhibition occurs only up to 10 minutes after CO treatment (Figure 5). One might speculate that this transient inhibition assures sufficient ROS generation to signal protective pathways, although not enough to induce damage. On the other hand, after 30 minutes COX activity is enhanced by CO treatment (Figure 5). Interestingly it is in accordance to our previous data showing an increase on ATP/ADP translocase activity of ANT [21] or a mitochondrial hyperpolarization by low concentrations of CO [20]. In summary, low doses of CO appear to accelerate mitochondrial oxidative phosphorylation and oxygen consumption. Another hypothesis to be considered is whether transient inhibition of COX activity also decreases calcium uptake protecting mitochondria against MMP.

Further studies are needed to elucidate the mechanisms implicated in ROS signalling, in particular how CO modifies and/or accelerates mitochondrial oxidative phosphorylation and oxygen consumption.

## Conclusions

Thus, for the first time, it was demonstrated that CO inhibits MMP in isolated liver mitochondria, by preventing mitochondrial swelling, mitochondrial depolarization and the opening of a non-specific pore through inner membrane. Additionally, small amounts of ROS generation are essential for signalling MMP inhibition by CO. In conclusion, it can be hypothesized that part of the CO's anti-apoptotic property in hepatocytes and/or liver is due to its capacity to limit mitochondrial membrane permeabilization, preventing the release of pro-apoptotic factors into the cytosol.

## Methods

### Isolation of mouse liver mitochondria

Mitochondria were isolated from mouse liver (C57, female, 6-12 week old, Instituto Gulbenkian de Ciência, Portugal) by differential centrifugation and purified on Percoll gradient, according to [22]. Mitochondrial protein was quantified using BCA assay (Pierce, Illinois). All mitochondrial assays were performed under atmospheric air, without oxygen level control.

### Preparation of CO solution

Fresh stock solutions of CO gas were prepared daily and carefully sealed immediately after. PBS (Phosphate Buffered Saline) was saturated by bubbling 100% of CO gas during 30 minutes to produce 10^-3 ^M stock solution. The concentration of CO in solution was determined spectrophotometrically, as previously described [23]. CO compressed gas at 100% was purchased from Linde, Germany.

### Measurement of ROS generation

ROS generation was monitored by the conversion of 2',7'-dichlorofluorescein diacetate (H_2_DCFDA, Invitrogen, UK) to fluorescent 2', 7'-dichlorofluorescein (DCF). 25 μg of mitochondrial protein was incubated with 5 μM of H_2_DCFDA and 10, 50, 100 or 250 μM of CO or 500 μM of hydrogen peroxide, in swelling buffer. Fluorescence (λ_exc_: 485 nm, λ_em_: 530 nm) was measured using Biotek Synergy 2 Spectrofluorimeter during 30 minutes at 37°C. ROS generation was calculated as an increase over baseline levels, determined for untreated cells and considering 100% of ROS generation with 500 μM of hydrogen peroxide. In some cases, β-carotene (1 μM) was added to isolated mitochondria 10 minutes prior CO treatment.

### Swelling and depolarization assays

25 μg of mitochondrial protein was diluted in swelling buffer for swelling (decrease in optical density at 540 nm) or depolarization rhodamine 123 (1 μM) fluorescence dequenching assay containing or not 10 μM of CO for 15 min of incubation at room temperature, as described in [16]. In some cases, β-carotene (1 μM) was added to isolated mitochondria 10 minutes prior CO treatment.

Mitochondrial swelling was assessed by the decrease in optical density at 540 nm measured for 30 minutes at 37°C, using Biotek Synergy 2 Spectrofluorimeter. 100% of swelling is calculated based on the optical density decrease between non-treated and 12.5 μM Ca^2+ ^or 300 μM atractyloside treated mitochondria.

For depolarization assessment by Rhodamine 123 dequenching, 6.25 μM of Ca^2+ ^or 300 μM of atractyloside were added. The fluorescent measurements (λ_exc_: 485 nm, λ_em_: 535 nm, Biotek Synergy 2 Spectrofluorimeter) were followed at 37°C and are expressed in percentage relative to the positive control 5 μM of Ca^2+ ^or 300 μM atractyloside (100%) at the indicated time point, as described in [16].

### Inner membrane permeabilization assay

Citrate synthase activity assay was used to assess the inner membrane permeability according to [16]. Upon inner mitochondrial membrane permeabilisation acetyl-CoA is able to enter into mitochondrial matrix, reacting with citrate synthase. 5, 5'-dithio-bis 2-nitrobenzoic acid (DTNB) and deacetyled acetyl-CoA reaction gives 5-thio-2-nitrobenzoate (TNB) which can be followed by absorbance at 412 nm. Briefly, 25 μg of protein from isolated mitochondria was incubated with CO (10 μM) in swelling buffer containing 100 μM of DTNB, 300 μM of acetylCoA and 1 mM of oxaloacetate. Inner membrane permeabilisation was induced by atractyloside at 300 μM or Ca^2+ ^at 5 μM. Whenever the case, β-carotene (1 μM) was was added 10 minutes prior CO treatment. The absorbance at 412 nm was acquired for 20 minutes, using Biotek Synergy 2 Spectrofluorimeter. For slope calculation, absorbance values for each different condition were normalized by the absorbance value corresponding to the control non-treated mitochondria.

### Cytochrome c oxidase activity assay

Cytochrome c oxidase (COX) activity was determined using a kit from Sigma CYTOCOX1. It is a colorimetric assay based on the oxidation of ferrocytochrome c to ferricytochrome c by COX. The reaction can be followed by a decrease in the absorbance at 550 nm, at 25°C. Briefly, mitochondria were treated with 10 μM of CO at 37°C for 5, 10, 15 and 30 minutes. The absorbance at 550 nm was acquired using Spectrophotometer DU-530, under the following conditions: 230 μg of mitochondrial protein was incubated with 0.45 mM Tris-HCl containing 12 mM of sucrose, 9 mM Tris-HCl containing 100 mM KCl and 0.01 mM of ferrocytochrome c, during 1 minute, with 10 seconds of interval. For each sample, COX activity was expressed in mU/mL.

### Statistical analysis

Mitochondrial data is presented as a representative result of at least three independent batchs or assays. All values are mean ± SD, n ≥ 3. Error bars, corresponding to standard deviation, are represented in the figures. Statistical comparisons were performed using ANOVA: single factor, with *p *< 0.05, n ≥ 3. *p *< 0.05 means that samples are significantly different at a confidence level of 95%.

## List of Abbreviations

CO: carbon monoxide; HO: heme-oxygenase; ROS: reactive oxygen species; MMP: mitochondrial membrane permeabilization; Atra: atractyloside; ΔΨm: mitochondrial membrane potential; H_2_DCFDA: 2',7'-dichlorofluorescein diacetate; β-c: β-carotene; RT: room temperature; COX: cytochrome c oxidase

## Authors' contributions

CSFQ has made the acquisition and interpretation of data and drafted the manuscript; ASA performed experiments important for the re-estructure of the manuscript; PMA has been involved in revising the manuscript; CB has made contributions to conception of the manuscript and to revise it critically and HLAV conceived of the study, participated in its design, coordination and acquisition of data, and helped to draft the manuscript. All authors read the paper and approved the final manuscript.
